# P-1334. Assessment of Immunity Gaps and Determination of Optimal Age for Measles-Containing Vaccine Booster Dose in Measles-Endemic Country

**DOI:** 10.1093/ofid/ofae631.1512

**Published:** 2025-01-29

**Authors:** Thundon Ngamprasertchai

**Affiliations:** Faculty of Tropical Medicine, Mahidol University, Bangkok, Krung Thep, Thailand

## Abstract

**Background:**

Despite highly measles immunized countries, immunity gaps in adolescents and young adults are a key issue posing an obstacle to measles elimination. This study aims to identify the gaps by estimating the probability of age-stratified seropositivity and optimal age for measles-containing vaccine (MCV) booster administration to effectively fill the gaps in an endemic country.

**Figure:**
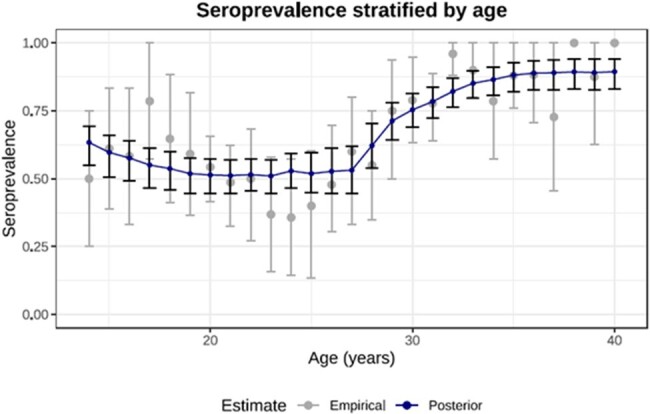

Posterior (blue) vs empirical (gray) age-stratified seroprevalence curves. Posterior error

bars correspond to 95% credible intervals, while empirical error bars indicate 95% confidence intervals

generated using bootstrap resampling over 1000 iterations.

**Methods:**

We retrospectively obtained measles serological results among individuals aged 13 to 39 years from multi-centre hospital between January 2018 and December 2021 in Thailand. We developed a dynamic probability model, stratifying seropositivity due to vaccination or natural infection. We calibrated the model to age-stratified seropositivity data within a Bayesian setting using the Metropolis-Hastings algorithm. A scenario analysis to determine the most effective age for MCV booster administration was also performed.

**Figure:**
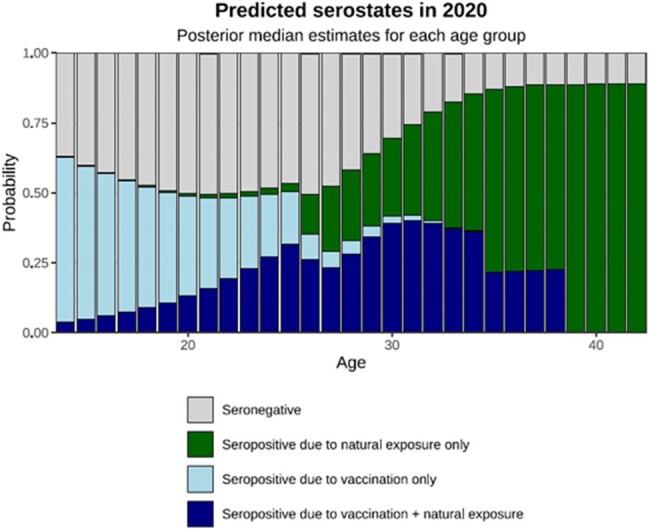

Posterior median estimates for individual serostatus in the year 2020, stratified by age.

**Results:**

The overall prevalence of measles seropositivity was 65.6% (N=543). Young adults (aged 21 to 30 years) displayed the lowest seropositivity at 59.3% (N=208) while individuals aged 31 to 39 years demonstrated the highest seropositivity at 87.3% (N=158). The average duration of vaccine induced immunity was estimated to be 16.0 years (95% credible interval (CI), 11.1 to 21.0 years); while natural exposure immunity was estimated to be lifelong. Posterior curves for the age-stratified seroprevalence (Figure 1A) exhibited a decreasing trend from ages 14 to 20 years but an upward trend from 26 to 30 years. The relative contribution of vaccine or natural exposure caused seropositivity was estimated for individuals stratified by age in the year 2020 (Figure 1B). The age at which a given individual’s serostatus reached a 50% probability of negativity was found to be approximately 20 years depending on measles circulation (Figure 1C).

**Figure:**
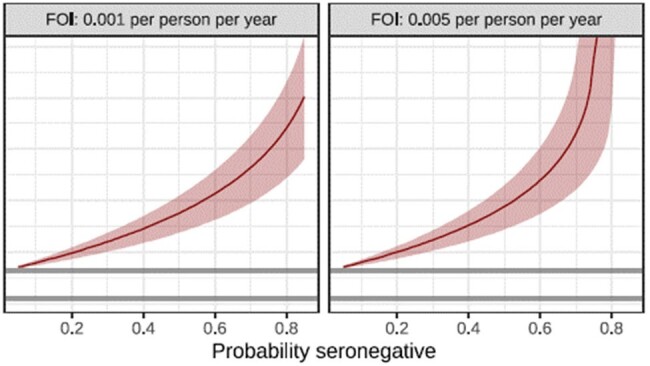

Age at which a given seronegativity threshold is reached. Posterior medians (based on 1000

parameter combinations sampled uniformly at random without replacement) are shown with solid lines;

ribbons indicate 95% credible intervals.

**Conclusion:**

Our findings highlight young adults aged 21 to 30 years as significant gaps in measles immunity, posing an increased risk of transmission. A single MCV booster dose at the age of 20 years potentially closes the gap and enhances measles elimination programmes.

**Disclosures:**

**All Authors**: No reported disclosures

